# Pre-mRNA processing factor 3 enhances the progression of keratinocyte-derived cutaneous squamous cell carcinoma by regulating the JAK2/STAT3 pathway

**DOI:** 10.1038/s41598-020-65928-8

**Published:** 2020-06-01

**Authors:** Siyao Zuo, Xin Li, Wanguo Bao, Shanshan Li

**Affiliations:** 1 Department of Dermatology and Venereology, The First Hospital of Jilin University, Changchun, China; 2 Department of Infectious Disease, The First Hospital of Jilin University, Changchun, China

**Keywords:** Metastasis, Oncogenesis

## Abstract

The precise role of pre-mRNA processing factors (PRPs) in human tumorigenesis has not been yet explored. The object of the present study was to explore the effects of PRP3 in a common metastatic skin cancer, keratinocyte-derived cutaneous squamous cell carcinoma (cSCCs). RT-qPCR and western blotting were conducted to measure the expression levels of PRP3 in various cSCC cell lines and cSCC tissues. A benign epidermal keratinocyte cell line was transfected with a eukaryotic expression plasmid to overexpress PRP3. In addition, the endogenous expression level of PRP3 in cSCC cells was silenced using a short hairpin RNA method, and the role of PRP3 on cell proliferation and migration was examined by Cell Counting Kit-8, colony formation, wound healing assay and Transwell assays following knockdown in cSCC cells, and overexpression in keratinovcyte cells. Elevated levels of PRP3 mRNA and protein were noted in cSCC cell lines or cSCC tissues compared with actinic keratosis (AK) or benign epidermal keratinocyte cell line, respectively. Upregulation of PRP3 expression was found to be associated with poor clinical outcomes in patients with cSCCs. The upregulation of PRP3 promoted cell viability, metastasis and the activity of the JAK2/STAT3 pathway in epidermal keratinocyte cells. Interestingly, loss of PRP3 had no obvious impact on cell viability and migration in benign epidermal keratinocyte cells. Functionally, the inhibition of the JAK2/STAT3 pathway reversed the increased cell viability and migration of cSCC cells induced by PRP3. Taken together, the present observations indicated that PRP3 served as a tumor active factor in cSCCs by targeting the JAK2/STAT3 pathway. Moreover, it is implied that impeding the PRP3 activity may selectively constrain cancer cell growth and migration with limited effect on normal skin cells.

## Introduction

In mammals, ~95% of the nucleotides in the primary transcript (pre-mRNA) of a protein-encoding gene are introns^1^. These introns were deleted exactly by splicing before the mRNA can be transported from the nucleus into the cytoplasm, where it can be translated^2^. It is becoming increasingly clear that alternative splicing is a fundamental component of eukaryotic gene regulation, influencing cell differentiation, development and many procedures in the nervous system^3^. Alternative splicing also momentously enlarges the gene coding ability and >60% of human genes are alternatively spliced ^4,5^. Introns are removed through two transesterification reactions catalyzed by the spliceosome^3^. The spliceosome comprises five smalls nuclear RNAs (snRNAs), such as U1, U2, U4, U5 and U6 snRNAs, which form five small nuclear ribonucleoproteins (snRNPs) with their related proteins, along with abundant other protein splicing factors ^6,7^. The formation of the E-complex involves the initial recognition of an intron by the spliceosome^3^. The 5′ ss is recognized by U1 snRNP, whereas the branch point sequence (BPS) and polypyrimidine tract (PYT) interact with other splicing factors. Subsequently, the U2 snRNP joins the spliceosome to form the a complex, which is followed by the enrollment of the U4/U6.U5 triple snRNP (tri-snRNP), forming the B complex^8^. Extensive structural reorganizations happen at this stage to formula the catalytically active B complex that mediated the first splicing reaction^9^. After the first step reaction, the spliceosome repositions the substrate, allowing the second catalytic reaction and forming the C complex^10^. The second step is followed by post-catalytic rearrangements to release the mature mRNA for the nuclear export, releasing the lariat intron, which will be degraded, and the snRNPs, which will be recycled^11^.

Disorders in splicing lead to >30% of human genetic disorders, including retinitis pigmentosa (RP), spinal muscular atrophy and myotonic dystrophy^12^. RP is an autosomal dominant genetic disorder that leads to photoreceptor degeneration and vision impairment^13^. Mutations or deletions of various splicing factors, including PRP3, small nuclear ribonucleoprotein U5 subunit 200 (Brr2) and PRP31, have been found to cause various subtypes of RP^14^. These proteins are all constituents of the U4/U6.U5 tri-snRNP complex and are ubiquitously expressed in all tissues^15^. Intriguingly, mutations or heterozygous deletion of these splicing factors affect primarily photoreceptors, which are one of the most metabolically active cell types in the body, and have no obvious effect on any other organs^16^. Furthermore, a 90% reduction in the protein level of splicing factor 3b subunit 1 (SF3b1), a key component of the U2 snRNP complex, contributes to developmental defects in high specific organs rather than lethality or widespread disorders in many organs, indicating the cell type-specific impacts of suppressing the basal splicing machinery^17^. Therefore, the present study assumed that constraining the spliceosomal activity may selectively constrain tumor cell growth or survival with limited effect on normal cells.

Keratinocyte-derived cutaneous squamous cell carcinoma (cSCCs) is the most common metastatic skin cancer, and its incidence is augment owing to augmented sunlight exposure and the elderly of population^18^. Therapies intended to control the metastasis and recurrence of cSCCs has been found to be insufficient in the treatment of this disease, and cSCCs in immunosuppressed patients was appeared to be associated with higher ratio of mortality^19^. Our present study identified that PRP3 was upregulated in cSCCs; however, to the best of our knowledge, there are no studies investigating the impact of PRP3 on the tumorigenesis of malignant cSCCs. The JAK2/STAT3 signaling pathway, a classical signal transduction pathway, plays a key role in controlling lots of aspects of development and cell maintenance in human beings, such as cell proliferation, migration, invasion, apoptosis, and cell polarity^20^. A previous study reported that JAK2/STAT3 signal alteration has a vital impact on the tumorigenesis of skin cancer^21^. Currently, there is no evidence demonstrating the correlation between PRP3 and JAK2/STAT3 signal alteration in cSCCs. Therefore, the aim of the present study was to investigate the expression pattern of PRP3, its role and its underlying mechanisms in cSCCs malignancy. Importantly, the regulatory mechanism of the PRP3/JAK2/STAT3 axis in cSCCs remains unclear. Therefore, the dysregulation of PRP3 and its regulatory mechanism in cSCCs were examined in the present study. The present results may facilitate the development of a novel and efficient cSCCs treatment.

## Materials and methods

### Experimental sample

In the present study, normal human skin (n = 16), actinic keratosis (AK) (n = 42), cSCCs *in situ* (n = 24) and sporadic cSCCs (n = 34) specimens were obtained from patients in Cancer Hospital of Jilin Province between May 2007 and July 2014. Before the experiment, written informed consent was collected from all the patients. The participants did not receive any treatment except for surgery. The present study was approved by The Institutional Ethics Committee of Cancer Hospital of Jilin Province.

### Cell lines and transfection

Human benign epidermal keratinocyte cell line (HaCaT), and three cSCC cell lines (A431, SCC13 and HS-1) were seeded in DMEM containing 10% FBS. All cells were cultured at 37 °C in 5% CO_2_. PRP3 vector and control vector were bought from Shanghai Genechem Co., Ltd. PRP3 vectors were transfected into cSCC cells and using Lipofectamine 2000 (Invitrogen; Thermo Fisher Scientific, Inc.) following the manufacturer's instructions. G418 (Sigma-Aldrich; Merck KGaA) was used to expand G418-resistant clones in culture as a monoclonal population.

### JAK2 inhibitor treatment

The JAK2 inhibitor AG490 was diluted to a final concentration of 40 µM in DMSO and stored at −20 °C, cells were subsequently treated for 24 h at 10 nM in order to efficiently inhibit JAK2. Cells treated with the same volume of DMSO served as the control group.

### RNA extraction and reverse transcription-quantitative PCR (RT-qPCR)

Total RNA was extracted using TRIzol reagent (Invitrogen; Thermo Fisher Scientific, Inc.) as previously described^22^. The cDNA was synthesized by PrimeScript RT reagent (Takara Bio, Inc.). RT-qPCR was performed using SYBR Green Master Mix II (Takara Bio, Inc.) according to the manufacturer’s instructions. The expression levels of PRP3 and PRP31 were normalized to GAPDH. The expression levels of the genes investigated were calculated using the 2-∆∆Cq method. The primers used in the present work were as follows: PRP3 forward, 5′-GAGAATGCGAAGGAACAAGC-3′ and reverse, 5′-AGTCTTGCCGCTGTAGGTAA-3′; PRP31 forward, 5′-GGATCCATGTCTCTGGCAGATGAGCTCTTA-3′ and reverse, 5′-CCGCGGTCAGGTGGACATAAGGCCACTCTT-3′; GAPDH forward, 5′-ACATCGCTCAGACACCATG-3′ and reverse, 5′-TGTAGTTGAGGTCAATGAAGGG-3′.

### Western blot analysis

Cells were lysed using RIPA buffer (Beyotime Institute of Biotechnology). Then, the supernatant containing the total protein was collected as previously described^23^. The protein was separated by 10% SDS-PAGE. The protein was blocked using 5% non-fat milk for 1 h. The membranes were incubated with the following primary antibodies: PRP3 (cat. no. # ab50386, Abcam), PRP31 (1:1,000 dilution; cat. no. #ab188577, Abcam), p-JAK2 (cat. no. #4406, Cell Signaling Technology, Inc.), JAK2 (cat. no. #4089, Cell Signaling Technology, Inc.), STAT3 (cat. no. #4904, Cell Signaling Technology, Inc.), p-STAT3 (Thr705) (cat. no. #52075, Cell Signaling Technology, Inc.), and β-actin (1:2,000 dilution; cat. no. #ab107061, Abcam). Primary antibodies were incubated with the membranes overnight at 4 °C. The diluted secondary antibodies were added to the membranes for 1 h. Finally, the protein was examined using an ECL reagent (EMD Millipore) and the immunoreactive bands analyzed with Image Lab 6.0.1 software (Bio-Rad Laboratories).

### Immunofluorescence

The cells were washed 3 times with PBS, fixed with 4% paraformaldehyde for 10 min at room temperature, permeabilized with 0.1% Triton X-100, and blocked in PBS with 2% bovine serum albumin for 1 h. The staining was performed with a rabbit anti-human PRP3 antibody (cat. no. # ab50386, Abcam). Images were obtained using an Olympus IX81 microscope with an MT20/20 illumination system.

### short hairpin RNA (shRNA) method

The packaging construct (pHelper 1.0), the (vesicular stomatitis virus G, VSVG) VSVG–expressing construct (pHelper 2.0), pGCSIL-EGFP plasmid, pGCSIL-scramble vector and pGCSIL PRP3-shRNA construct were purchased from Genechem Biotech Co., Ltd. The shRNA-mediated knockdown was performed as previously described^24^. HEK 293 T cells (at 70–80% confluence) maintained in 6-well dishes were transfected with the aforementioned constructs using Lipofectamine (cat. no. 11668027; Thermo Fisher Scientific, Inc.), according to the manufacturer's protocol. The viral stocks were concentrated via ultracentrifugation and dissolved in Hanks’ balanced salt solution. The cSCC cells and benign epidermal keratinocyte cells were transfected with the viral stocks at a multiplicity of infection of 200.

### Cell Counting Kit-8 (CCK8) assay

Transfected cells (4 × 10^3^ cells/well) were seeded in DMEM with 10% FBS for 12, 24, 36, 48, 60, 72 or 96 h. Next, the suspension of cells was incubated with 20 μl of CCK8 for 4 h. Then, 150 μl DMSO was added into the medium. After 10 min, cell viability was assessed using a microplate reader (Olympus Corporation) to determine the optical density at 490 nm.

### Colony formation assay

Briefly, stably transfected HACAT or SCC13 cells were plated in 6-well plates at 400 cells per well at 37 °C for 10 days. the cell colonies were washed with PBS twice, and fixed with methanol for 30 min, and dyed with 0.1% crystal violet diluted in PBS for 15 min. The colonies which contained more than 120 cells were counted.

### Transwell chamber assay

Transwell assay was used to assess cell invasion. Upper chambers were coated with Matrigel (BD Biosciences) to detect SCC13 or HaCaT cell invasion. The transfected cells (5 × 10^3^ cells/well) were seeded in the upper chamber, and the lower chamber was filled DMEM containing 10% FBS. The invasive cells were fixed and stained for 30 min. Finally, the invasive cells were fixed and stained for 30 min, and examined under a light microscope (Olympus Corporation).

### Wound healing assay

A cell monolayer scratch assay was performed as described previously^25^. Briefly, HACAT or SCC13 cells were seeded in 6 well plates at 3.5 × 10^5^ cells per well, grown to ~100% confluence and pretreated with mitomycin C (10 μg/ml) for 2 h to inhibit cell proliferation before scratching. A scratch wound was created using a 20 μl pipette tip and was imaged at the same position at 0, 12 and 24 h.

### Statistical analysis

Data are presented as the mean ± SD, which were analyzed using SPSS V17.0 and GraphPad Prism V5.02. Statistical analyses and graphical depictions were performed through SPSS V17.0 and GraphPad Prism V5.02 software. Chi-squared test, one-way ANOVA with Tukey’s post hoc test and Univariate Kaplan-Meier method with log-rank test were used to calculate differences between groups. P < 0.05 was considered to indicate a Statistically significant difference. All methods were performed in accordance with the relevant guidelines and regulations.

## Results

### The expression of PRP3 is increased in cSCC tissues

**Figure 1:**
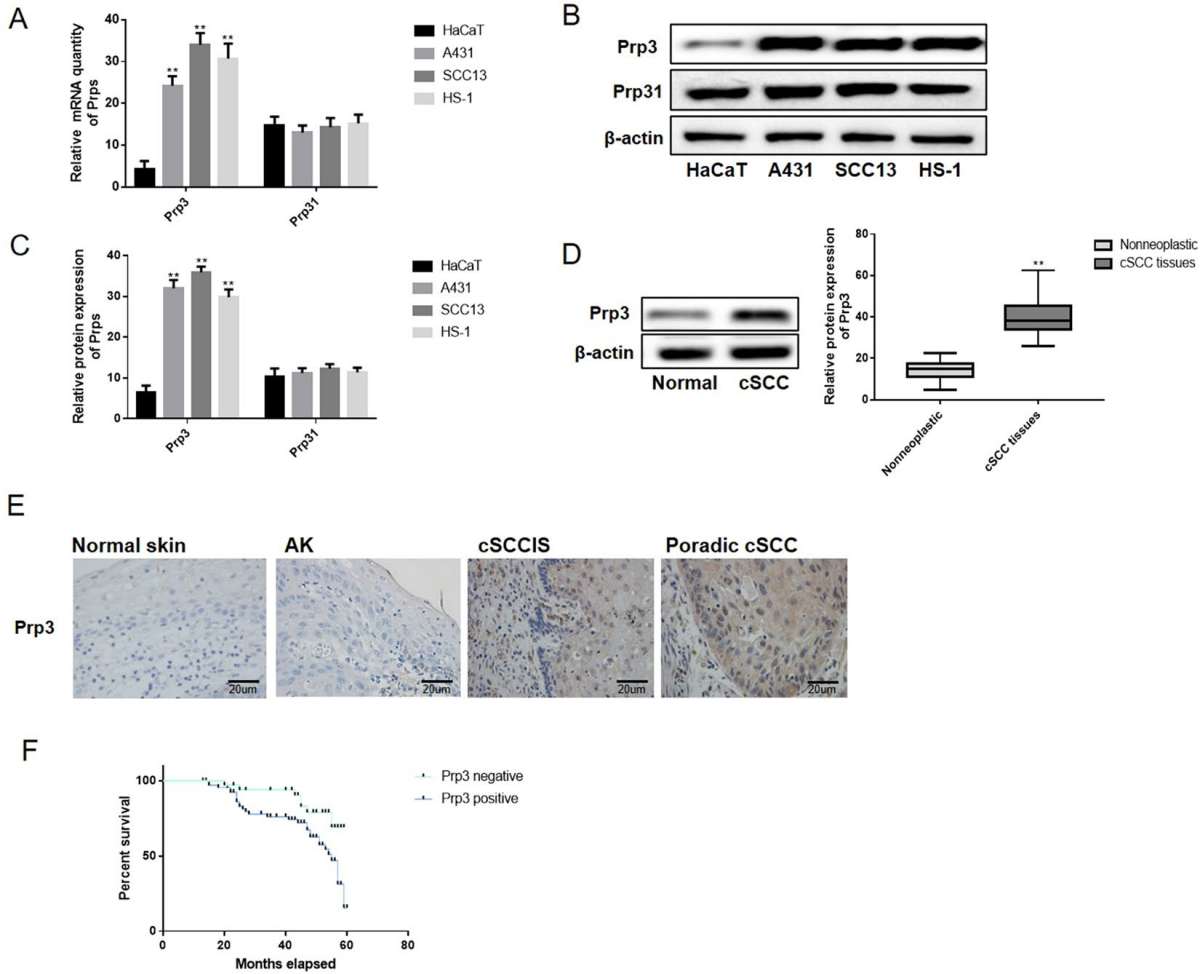
The expression of PRP3 is increased in cSCC cells and tissues. (**A**) PRP3 mRNA expression in cSCC cells compared with benign epidermal keratinocyte cells. (**B**) PRP3 protein expression in cSCC cells compared with benign epidermal keratinocyte cells. (**C**) Relative protein expression of PRP3 in cSCC cells versus benign epidermal keratinocyte cells. (**D**) PRP3 protein expression in cSCC tissues was examined via western-blotting. (**E**) PRP3 protein expression in normal human skin, AK, cSCCs *in situ* and sporadic cSCCs tissues were explored via IHC. (**F**) High PRP3 expression is associated with poor prognosis in cSCCs patients. ^*^P < 0.05, ^**^P < 0.01. PRP3, pre-mRNA processing factor 3.

The alternation of PRP3 and PRP31 expression was detected in cSCC cells. RT-qPCR and western blotting showed that the expression of PRP3 was increased in SCC13, A431 and HS-1 cells compared with HaCaT cells (P < 0.01; Fig. 1A–C), although there were no differences in PRP31 expression levels among HaCaT, SCC13, A431 and HS-1 cells. The expression of PRP3 in normal human skin (n = 16), actinic keratosis (AK) (n = 42), cSCCs *in situ* (n = 24) and sporadic cSCCs (n = 34) specimens were explored via IHC method. Similarly, PRP3 expression was found to be higher in cSCC tissues compared with AK tissues (P < 0.01; Fig. 1D,E and Table 1). In addition, the correlation between abnormal PRP3 expression and clinical features in cSCCs patients was investigated. As shown in Table 1, the dysregulation of PRP3 was associated with tumor node and metastasis (TNM) stage (P < 0.01) and distant metastasis (P < 0.05). Furthermore, patients with cSCCs presenting high PRP3 expression showed a shorter overall survival time, indicating that upregulation of PRP3 predicted poor prognosis in patients with cSCCs (P < 0.01, Fig. 1F). The present results indicated that PRP3 may function as an important regulator of the pathogenesis of cSCCs.

**Table 1:** Expression of PRP3 and the clinicopathological characteristics in cSCCs patients.

Item	n	PRP3 (+)	PRP3 (−)	*P*
cSCC tissues	58	45	13	<0.01
AK	42	10	32	
Age (years)				
≤60	28	22	6	0.916*
>60	30	23	7	
Distant metastasis				
+	24	16	8	<0.05
−	34	29	5	
TNM stage (AJCC)				
I~II	26	16	10	<0.01
III~IV	32	29	3	

^*^No statistical significance was found with the Chi-square test/Chi-Square Goodness-of-Fit Test. TNM, tumor node and metastasis; AJCC, American Joint Committee on Cancer.

### Overexpression of PRP3 promotes cell viability and migration of human benign epidermal keratinocyte cell line

**Figure 2:**
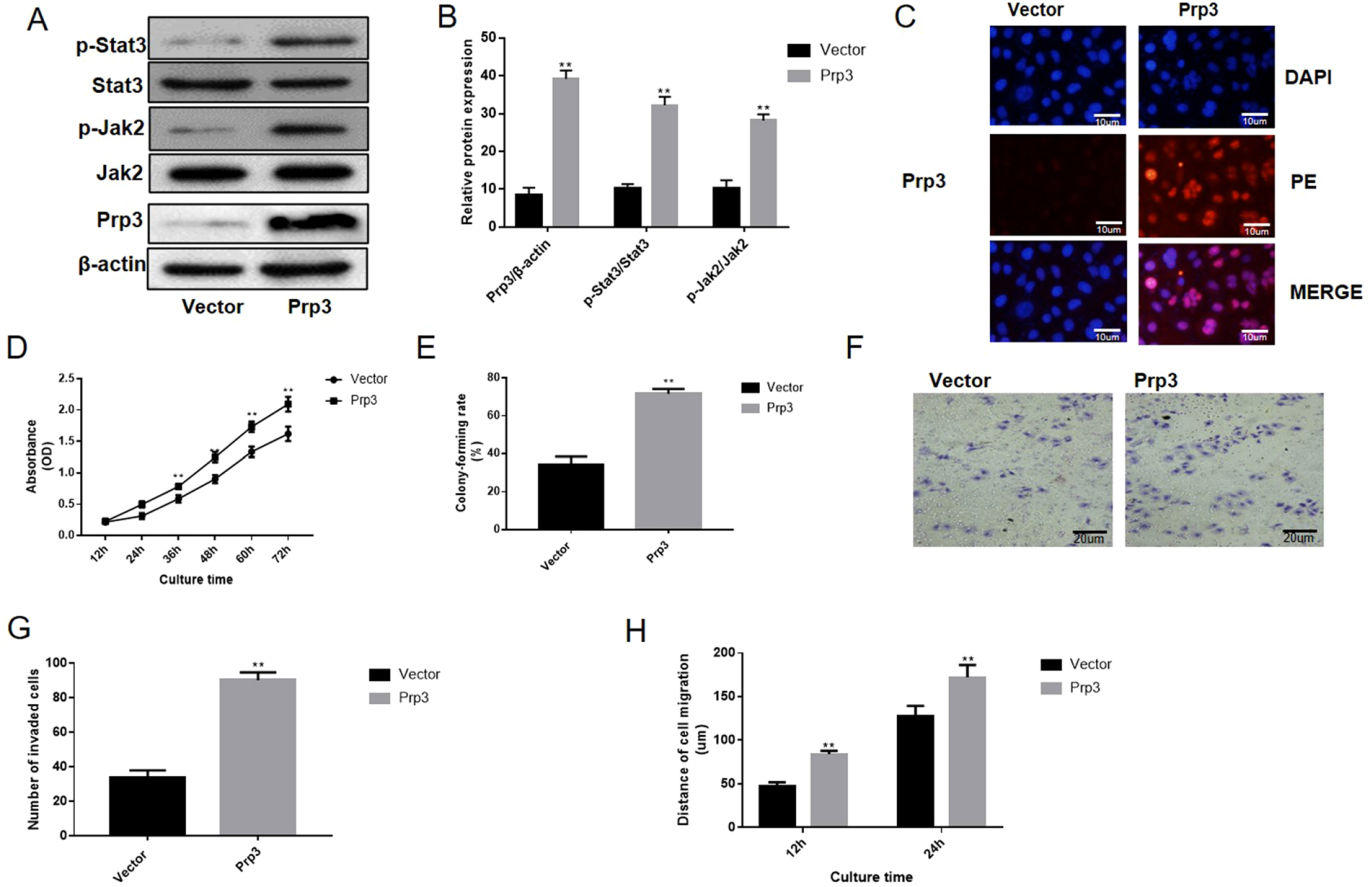
Upregulation of PRP3 promoted cell viability and migration in a human benign epidermal keratinocyte cell line. (**A**) The effect of PRP3 on the JAK2/STAT3 pathway was investigated. (**B**) The relative protein expression of PRP3, phosphorylated JAK2 and phosphorylated STAT3. (**C**) The expression location of PRP3 was explored via immunofluorescence (**D**) CCK8 assay revealed that impact of PRP3 on cell proliferation. (**E**) Colony formation assay revealed that overexpression of PRP3 promoted cell viability. (**F**) Transwell assay was used to explore the impact of PRP3 on cell invasion. (**G**) The analyze of the invaded cell number. (**H**) The ability of cell migration was explored via wound healing assay. ^*^P < 0.05, ^**^P < 0.01. PRP3, pre-mRNA processing factor 3.

PRP3 was overexpressed in human benign epidermal keratinocyte cell line HaCaT to perform a gain-of-function experiment. PRP3 expression was upregulated after overexpression in HaCaT cells (P < 0.01, Fig. 2A,B). The effect of PRP3 on the JAK2/STAT3 pathway was investigated to further examine its role in cSCCs. PRP3 overexpression was found to promote the expression level of phosphorylated JAK2 (P < 0.01) and phosphorylated STAT3 (P < 0.01) in HaCaT cells (Fig. 2A,B). Besides, immunofluorescence data discovered that PRP3 was mainly expressed in the cell nucleus (Fig. 2C). CCK8 and colony formation assay revealed that overexpression of PRP3 promoted cell proliferation and viability in HaCaT cells (P < 0.01, Fig. 2D,E). Transwell and wound healing assay showed that cell migration and invasion were increased following PRP3 overexpression in HaCaT cells (P < 0.01, Fig. 2F,H). Collectively, PRP3 promoted the proliferative, migratory and invasive abilities of epidermal keratinocyte cells.

### Loss of PRP3 has an inhibitory effect on cell viability, migration and invasion in cSCC cells

**Figure 3:**
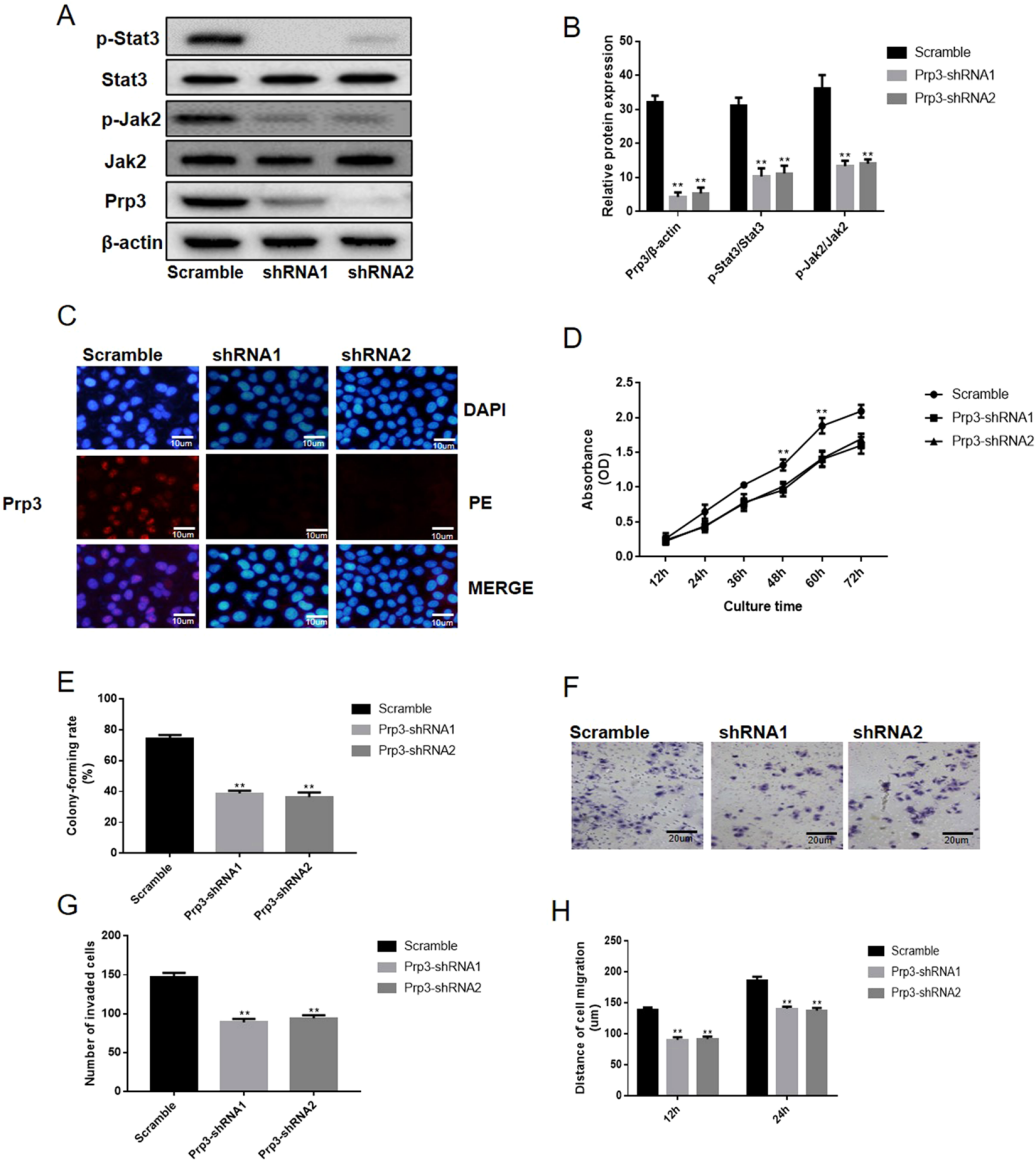
Loss of PRP3 has an inhibitory effect on cell viability and metastasis in cSCC cells. (**A**) PRP3 expression and the JAK2/STAT3 pathway were inhibited following PRP3 knockdown in SCC13 cells. (**B**) The relative protein expression of PRP3, phosphorylated JAK2 and phosphorylated STAT3. (**C**) The expression location of PRP3 was explored via immunofluorescence (**D**) CCK8 assay revealed that loss of PRP3 reduced cell proliferation. (**E**) Colony formation assay revealed that the loss of PRP3 inhibited the ability of colony formation. (**F**) Transwell assay showed that cell invasion was inhibited by PRP3 knockdown in SCC13 cells. (**G**) The analyze of the invaded cell number. (**H**) The ability of cell migration was explored via wound healing assay. ^**^P < 0.01. PRP3, pre-mRNA processing factor 3.

SCC13 cells were selected for further functional assay due to the high expression level of PRP3 in this cell type. PRP3 was silenced in SCC13 cells to perform a loss-of-function experiment. Western-blot and immunofluorescence assay revealed that PRP3 expression was decreased following PRP3 knockdown in SCC13 cells (P < 0.01, Fig. 3A–C). PRP3 knockdown was found to inhibit the expression level of phosphorylated JAK2 (P < 0.01) and phosphorylated STAT3 (P < 0.01) in SCC13 cells (Fig. 3A,B). CCK8 and colony formation assay revealed that loss of PRP3 reduced cell proliferation and viability in SCC13 cells (P < 0.01, Fig. 3D,E). Similarly, knockdown of PRP3 reduced cell invasion cells (P < 0.01, Fig. 3F,G). Wound healing assay showed that cell migration was inhibited following PRP3 knockdown in SCC13 cells (P < 0.01, Fig. 3H). Taken together, PRP3 knockdown reduced the proliferative, migratory and invasive abilities of cSCC cells.

### AG490 treatment reversed the carcinogenesis effect of PRP3 in benign epidermal keratinocyte cells

**Figure 4:**
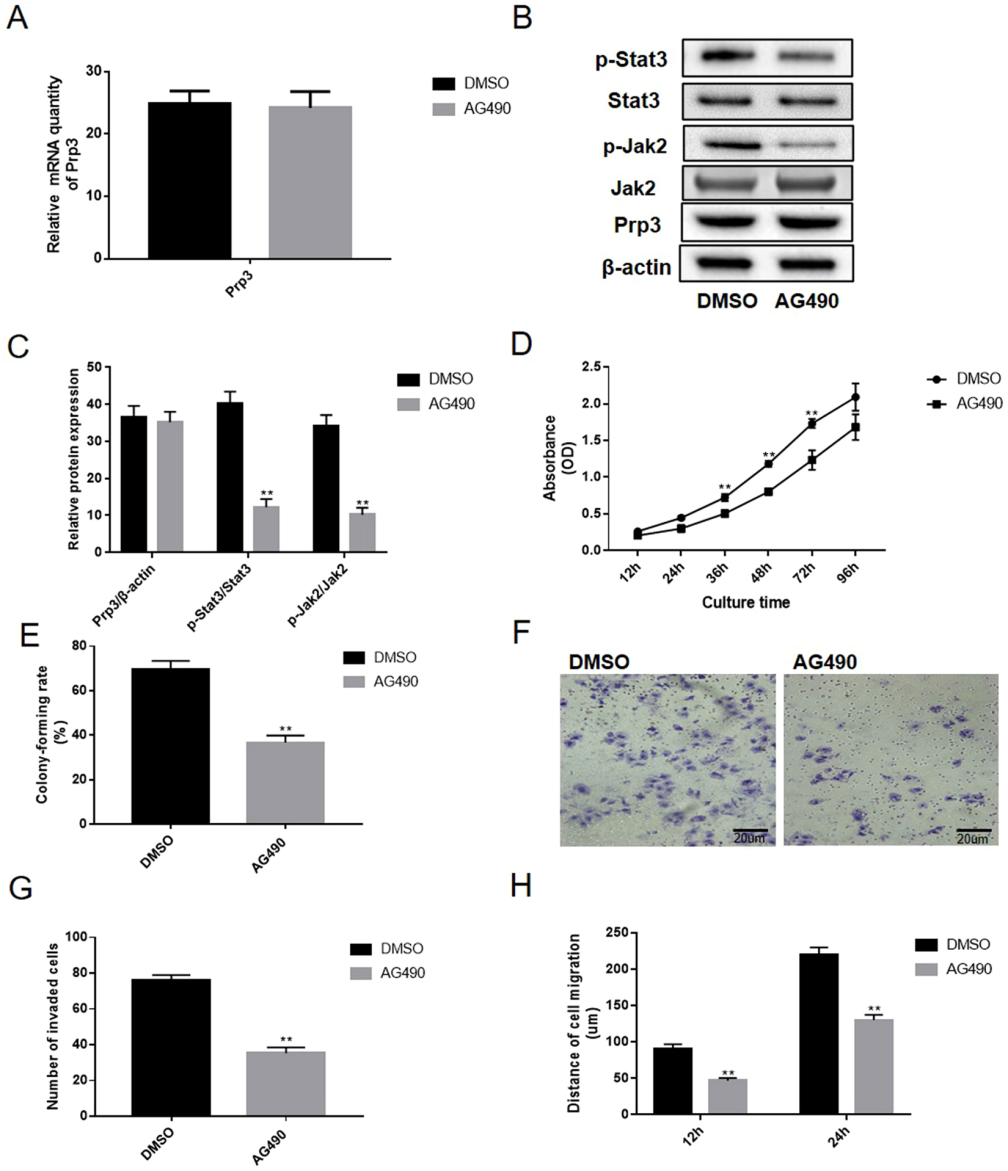
AG490 treatment reverses the carcinogenic-inducing effects of PRP3 in benign epidermal keratinocyte cells. (**A**) The impact of AG490 treatment on the PRP3 mRNA expression. (**B**) AG490 treatment inhibited the activation of JAK2/STAT3 pathway in benign epidermal keratinocyte cells. (**C**) The relative protein expression of PRP3, phosphorylated JAK2 and phosphorylated STAT3. (**D**) The AG490 treatment suppressed the promoted effects of PRP3 on cell proliferation. (**E**) Colony formation assay revealed that the AG490 treatment inhibited the ability of colony formation. (**F**) Promoted effect of PRP3 on cell invasion were inhibited by treatment with AG490. (**G**) The analyze of the invaded cell number. (**H**) The ability of cell migration was explored via wound healing assay. ^**^P < 0.01. EMT, epithelial*-*mesenchymal transition; PRP3, pre**-**mRNA processing factor 3.

Functionally, the effect of PRP3 on JAK2/STAT3 pathway in benign epidermal keratinocyte cells was abolished following treatment with 10 nM JAK2 tyrosinase inhibitor AG490 for 24 h. Our data revealed that AG490 treatment has no obvious impact on PRP3 mRNA expression (Fig. 4A). In addition, the effect of AG490 on the JAK2/STAT3 pathway was also identified in HaCaT cells (Fig. 4B,C). The AG490 treatment suppressed the cell proliferation and viability in HaCaT cells (Fig. 4D,E). Similarly, the effect of PRP3 on HaCaT cell invasion (P < 0.01, Fig. 4F,G) and migration (P < 0.01, Fig. 4H) were also inhibited following treatment with AG490. Collectively, AG490 treatment reversed the carcinogenic effects of JAK2/STAT3 pathway in benign epidermal keratinocyte cells. In summary, PRP3 was identified to serve a carcinogenic role via JAK2/STAT3 pathway in cSCCs progression.

### Loss of PRP3 has no obvious impact on cell viability in benign epidermal keratinocyte cells

**Figure 5:**
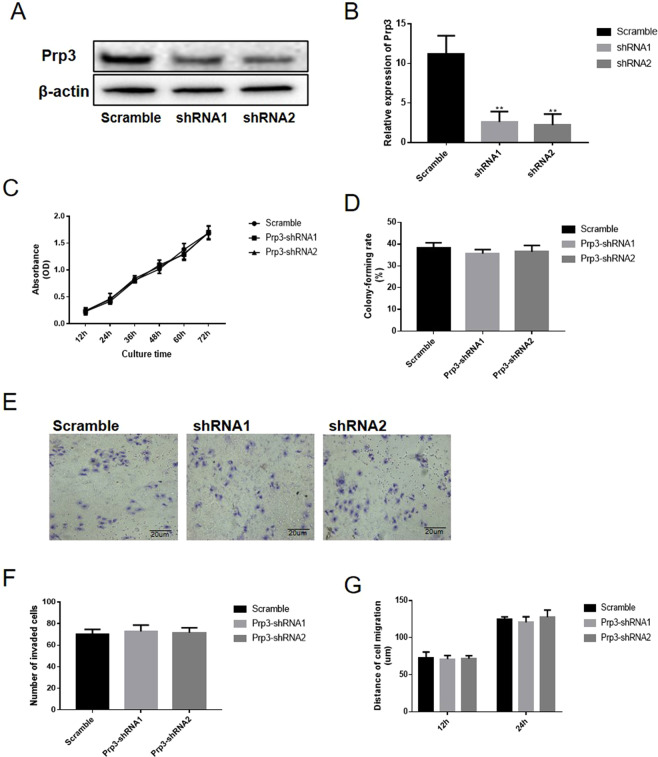
Loss of PRP3 has no obvious impact on cell viability in benign epidermal keratinocyte cells. (**A**) PRP3 was silenced in HaCaT cells. (**B**) The relative expression of PRP3 in HaCaT cells. (**C**) CCK8 assay revealed that loss of PRP3 had no obvious impact on cell viability in HaCaT cells. (**D**) The impact on the ability of colony formation was explored via colony formation assay. (**E**) Transwell assay showed that cell invasion was not obviously affected by PRP3 knockdown in HaCaT cells. (**F**) The analyze of the invaded cell number. (**G**) Wound healing assay showed that cell migration was altered by PRP3 knockdown in HaCaT cells. PRP3, pre-mRNA processing factor 3.

PRP3 was silenced in HaCaT cells to perform a loss-of-function experiment. PRP3 expression was reduced following PRP3 knockdown in HaCaT cells (P < 0.05, Fig. 5A,B). CCK8 assay revealed that loss of PRP3 had no obvious impact on cell proliferation in HaCaT cells (P > 0.05, Fig. 5C). Colony formation assay revealed that the PRP3 knockdown have no obvious impact on the ability of colony formation in HaCaT cells (P > 0.05, Fig. 5D). Transwell assay (P > 0.05, Fig. 5E,F) and wound healing assay (P > 0.05, Fig. 5G) showed that cell migration and invasion were not altered following PRP3 knockdown in HaCaT cells. Collectively, PRP3 knockdown had no obvious impact on the proliferative, migratory and invasive abilities of benign epidermal keratinocyte cells.

## Discussion

Recent studies demonstrated that the splicing apparatus is a limiting factor and various pre-mRNAs may compete with each other when the availability of the splicing apparatus is limited^26,27^. Inhibition of basal spliceosomal activity may have limited effect on splicing in all cells, but normal cells may tolerate the slightly lowered spliceosomal activity^28^. Inhibition of the basal spliceosomal components may lead to increased competition for a limited amount of functional spliceosome and selectively affect alternative splicing events that contain sub-optimal splicing sites^29,30^. Therefore, inhibition of the basal splicing machinery can adversely affect cancer cells via changes in alternative splicing events that are critical to cancer cells. A number of natural products isolated from the fermentation broths of Pseudomonas spp. and Streptomyces spp. that have potent anti-tumor properties support this hypothesis^31^. The spliceosome contains multiple enzymes, including eight RNA helicases, one GTPase, and various prolylisomerases and kinases^29^. These enzymes and many protein interactions in the spliceosome may be inhibited by small molecules, and targeting these spliceosomal components may represent a unique approach for cancer therapy. The potential side effect of these splicing-targeted therapies on photoreceptors is not a significant concern due to the existence of blood-retinal barrier, which has a similar structure as the blood-brain barrier and can prevent most small molecules from penetrating the barrier^32^. These compounds were first identified due to their potent cytotoxic and cell cycle arresting effect in multiple tumor cell lines, as these molecules exhibit an *in vitro* IC50 in the low nM range, and significant anti-tumor activity in animal models^33^. Recent mechanistic studies found that these compounds bind most tightly to the SF3b complex, which contains five protein components, of the spliceosome in cellular extracts^6^. Although the exact binding partner of these compounds in SF3b remains to be determined, accumulating evidence suggested that they bind to the interface between the subunits of SF3b proteins^34^. These compounds may have more potent growth arresting and cytotoxic effects on cancer cells, with no apparent general toxic effects due to extensive inhibition of general splicing and gene expression. One analog of these compounds (E7107) is currently in Phase I clinical trial for treating solid tumors^35^.

Tumor cells may be much more susceptible to a reduction in spliceosomal activity due to the rapid proliferation and high metabolic demand of cancer cells^33^. This effect was identified for proteasome inhibitors, such as velcade, which have been successfully used for cancer therapy^36^. To date, suppression of PRP3 function has never been clinically targeted in cSCCs. In this present study, it was hypothesized that inhibition of other spliceosomal component in addition to SF3b can selectively inhibit cancer cell growth and survival with limited side effects on normal cells^37^. The present results on PRP3 knockdown, a component of the tri-snRNP, in cSCCs SCC13 cells support the hypothesis of the present study. The present results revealed that the loss of PRP3 had no significant impact on cell viability of benign epidermal keratinocyte cells, but significantly inhibited the viability and metastatic potential of cSCC cells. Moreover, the present observations suggested that PRP3 was upregulated in cSCC tissues and was associated with distant metastasis. In addition, the influence of PRP3 on the JAK2/STAT3 signaling pathway in benign epidermal keratinocyte cells was examined, and the present results suggested that PRP3 activated the JAK2/STAT3 signaling pathway. Furthermore, the present observations suggested that the inhibition of JAK2 /STAT3 signaling pathway induced a reduction of the metastatic potential of cSCC cells that stably expressed PRP3. In addition, knockdown of PRP3 in an cSCCs cell line (SCC13) leads to a suppression of the JAK2/STAT3 signaling pathway and reduced the metastatic potential of SCC13 cells.

## Study Limitations

Nevertheless, the detailed molecular mechanisms of the signal transduced from nuclear PRP3 to JAK2 require further investigation.

## Conclusions

In summary, the present study identified an upregulation in PRP3 in cSCCs, which was associated with poor prognosis in cSCCs patients. Functionally, the loss of PRP3 could specifically inhibit the cell viability and metastasis and activated JAK2/STAT3 in cSCC cells but not in human benign epidermal keratinocyte cells. Although the present study has preliminarily investigated the regulatory mechanism of PRP3, further studies on PRP3 in cSCCs are required.

## Data Availability

The datasets used and/or analyzed during the present study are available from the corresponding author on reasonable request.
